# Quantitative assessment of vascular density in diabetic retinopathy subtypes with optical coherence tomography angiography

**DOI:** 10.1186/s12886-021-01831-8

**Published:** 2021-02-12

**Authors:** Fariba Ghassemi, Kaveh Fadakar, Sahar Berijani, Ameneh Babeli, Alireza Gholizadeh, Siamak Sabour

**Affiliations:** 1 Eye research center, Farabi Eye Hospital, Tehran University of Medical Sciences, Qazvin Square, Tehran, 1336616351 Iran; 2 Retina & Vitreous Service, Farabi Eye Hospital, Tehran University of Medical Sciences, Tehran, Iran; 3 Department of clinical epidemiology, School of Health and Safety, Safety promotion and Injury prevention research centre, Tehran, Iran; 4 Department of clinical epidemiology, Shahid Beheshti University of Medical Sciences, Tehran, Iran

**Keywords:** Capillary plexus, Choriocapillaris, Diabetes, Diabetic retinopathy, Optical coherence tomography angiography, Vascular density

## Abstract

**Background:**

Quantitative assessment of vascular density (VD) of retinal and choriocapillaris (CC) in various stages of diabetic retinopathy (DR) using spectral domain optical coherence tomography angiography (SD OCTA).

**Methods:**

188 eyes of 97 participants were recruited in this cross-sectional study. The macular OCTA (3x3mm) scan was performed and the computer algorithm assessed VD at the level of superficial capillary plexus (SCP), deep capillary plexus (DCP) and CC.

**Results:**

All measured parameters were decreased in retinal VD at the more extreme stages of DR, with the exception of SCP foveal VD. There was a constant pattern of decrease in VD of CC from normal cases to cases of NDR and NPDR and then a slight increase occurred in the PDR stage but never touching the normal quantities. Age, fasting blood sugar, and years of diabetes mellitus were correlated with reduced VD in different segments. Multivariate linear regression analysis showed that best-corrected visual acuity (BCVA) was positively correlated with parafoveal VD at SCP and VD of foveal area at CC. VD of all subfields of macular area except foveal DCP VD showed reduced levels in diabetic macular edema (DME) patients compared to those without DME.

**Conclusions:**

The findings of the study endorse retina VD changes as a potential biomarker for DR development before retinopathy becomes clinically evident. It seems that parafoveal VD of SCP and foveal VD of CC are good biomarkers to predict VA in the diabetic patients.

## Background

Diabetic retinopathy (DR) is a degenerative neurovascular disease caused by activation of multifactorial pathologic mechanisms, which leads to microvascular abnormalities including microaneurysm development, capillary non-perfusion, vascular leakage, and neovascularization [1–3]. Microvascular changes and destructions, as the loss of pericytes and endothelial cells and capillary leaks and occlusions also occur in earlier DR stages [4–6]. The resulting ischemia induces upregulation of angiogenic signaling molecules, including vascular endothelial growth factor (VEGF) and erythropoietin [7], which increases vascular permeability and ultimately fosters proliferative diabetic retinopathy (PDR) [8].

Fluorescein angiography (FA) and indocyanine green angiography (ICGA) are the gold standard imaging modalities for imaging of microvascular abnormalities of retina and choroid in DR for years. However, a number of limitations, including their invasive nature, the risk of allergic reactions in patients with iodine or seafood allergy or idiosyncratic reaction, the cost, the long duration of imaging and the occasional shortage of fluorescein and ICG dyes, have raised the clinical need for a shift to newer imaging techniques. For these reasons, FA and ICGA are not routinely performed in the early stages of DR for the assessment of the retinal and choroidal vasculatures [9].

New noninvasive imaging modalities (as OCTA) may provide valuable information about microvascular changes, the perfusion status of the retina and the likelihood of retinopathy progression during the various stages of DR. OCTA uses the motion contrast provided by flowing erythrocytes to allow dye-free and volumetric visualization of the retinal and choroidal vasculatures at micron-scale resolutions [10–13]. This technology uses split-spectrum amplitude-decorrelation angiography (SSADA) algorithm to detect motion of erythrocytes in the capillaries [14]. OCTA delivers depth-resolved retinal vascular structure images, making it possible to differentiate superficial and deep capillary plexuses (SCP and DCP) of retina and also choriocapillaris (CC) [11, 15, 16]. Previous qualitative studies in DR have shown that OCTA is able to delineate retinal capillary nonperfusion with higher resolution than conventional FA [17–19].

Previous studies have shown that vascular density (VD) of parafovea in SCP and DCP decreases with significant FAZ enlargement in diabetic patients with DR, even in those with no diabetic retinopathy (NDR), compared to healthy subjects [20, 21]. There were no significant differences in the retinal thickness between control subjects and patients with NDR at the same time. Significantly reduced VD in the SCP and DCP in mild nonproliferative DR (NPDR) in comparison to control subjects has also been observed [22]. It seems that retinal vascular alterations precede structural changes in the retina. This may highly suggest a causal role of circulatory deficit in the development of DR. [20] Kim et al. have detected qualitatively decreasing capillary density, branching complexity, and progressively increasing average vascular caliber in eyes at different stages of DR. [23] Overall, depending on the method used and methodological differences and metabolic status of the patients studied, conflicting results of the blood flow in diabetic retinal vessels have also been reported both to decrease [24–26] and to increase [27–30].

In this cohort, the aim is the quantitative measurement of VD at foveal and parafoveal area (as a marker of macular perfusion) using OCTA in subtypes of DR including those with NDR, NPDR, and proliferative diabetic retinopathy (PDR) as a continuum, and to compare these findings with each other and the normal population. In addition, we evaluate the correlation of VD as an independent predictor of BCVA in diabetic patients.

## Methods

This prospective cross sectional study was performed between January 2015 and December 2019 at Farabi Eye Hospital, a tertiary university eye center in Tehran, Iran. The institutional ethics committee of Tehran University of Medical Sciences approved the research protocol and a written informed consent was obtained from the participants. The study adhered to the tenets of the Declaration of Helsinki. More than 10 eligible patients did not provide informed consent and were excluded from the study.

Naïve diabetic patients with a history of more than 10 years have been recruited and the control patients have been selected from healthy volunteers. Inclusion criteria were best-corrected visual acuity (BCVA) of 20/20 for normal cases and refractive error between − 3 and + 1 D spherical equivalent in all groups. The normal volunteers had to have no ocular and systemic disease. Exclusion criteria were significant media opacity preventing high-quality imaging, motion and blinking artifact on the images, poor quality images, previous focal or panretinal laser photocoagulation, intravitreal anti-VEGF, steroid and/or potentially retinotoxic or neurotoxic drugs consummation, optic neuropathy, any previous ocular and macular disease, previous surgeries other than uncomplicated phacoemulsification (more than 3 months), any inflammatory diseases or active or recent infectious disease (ocular and/or systemic), immunosuppressive drugs or biologic therapies, pregnancy, and uncontrolled hypertension.

Demographic characteristics and relevant laboratory tests such as fasting blood sugar (FBS) and total serum cholesterol level and presence of hypertension were documented. Hemoglobin A1C levels were not checked for all the cases. Best-corrected visual acuity (BCVA) was measured on a Snellen chart and expressed as the logMAR.

Subjects underwent thorough ophthalmic exam including slit lamp biomicroscopy and fundus examination. Intraocular pressure (IOP) was measured with Goldman applanation tonometry. Diabetic patients have been diagnosed based on the criteria of the American Diabetes Association and all were under treatment for diabetes. The diabetic patients were classified into three groups ranked in ascending order of DR severity: NDR, NPDR, and PDR, based on early treatment for diabetic retinopathy study (ETDRS) classification [3, 31].

### Acquisition of the images

Clinical examination and SD-OCT imagings were performed at the same day between 8:00 am and 2:00 pm. Two professional image readers (FG, SB) checked and assessed all of the OCT images. AngioVue OCTA imaging (RTVue XR Avanti; Optovue, USA- version: 2016.1.0.23- beta) using SSADA algorithm was performed [32, 33]. This instrument performs 70,000 A-scans per second (840-nm) to capture OCTA images of horizontal and vertical B-scans in transverse dimension to provide a 3 × 3 mm (304 × 304 pixels) image centered at the fovea. Scans with low quality (i.e., presence of blink or motion artifact) were repeated until good quality scans were achieved. Automated segmentation was utilized for defining SCP, DCP, and CC. The SCP was defined as area between 3 μm below to internal limiting membrane (ILM) and 15 μm below internal plexiform layer (IPL). The DCP was considered to be between 15 μm and 71 μm below IPL.

Automatic segmentation was fine-tuned manually where appropriate. Each macular OCT-A layer was subdivided into nine areas of interest—whole image (WI), fovea, parafovea, superior hemifield (SH), inferior hemifield (IH), temporal (T), superior (S), nasal (N), and inferior (I) for quantitative measurements of the vascular density of SCP, DCP, and CC. The foveal region was outlined as a central circle with a 120-pixel (1.2 mm) diameter, and the parafoveal region was delineated as a ring, by 91 pixels wide, surrounding the foveal region [34]. To calculate VD, the AngioVue Analytics software extracts a binary image of the blood vessels from the gray scale OCTA image, and then calculates the percentage of pixels occupied by blood vessels in the defined region [35]. Diabetic macular edema (DME) was defined as the central macular thickness (CMT) of more than 300 μm.

### Statistical methods

All quantitative variables were reported as mean with standard deviation after confirming normality of distribution with the Kolmogorov- Smirnov test. Non-normal distributed parameters are reported by median with the range. All statistical analyses were performed using statistical software (SPSS software Version 21; SPSS, Inc., Chicago, IL, USA). Kruskal-Wallis test and one-way analysis of variance (ANOVA) were performed for nonparametric and parametric comparison. Mann Whitney U test and post-hoc analysis (dunnett’s test) were used to compare choroidal thicknesses between groups. In this study collinearity for different variables was checked. *P* values less than 0.05 were considered statistically significant.

## Results

### General characteristics

A total of 188 eyes of 97 participants with the mean age of 56.5 ± 8.9 years (range: 25–80) were analyzed. Of these 41 (42.3%) were male and 56 (57.7%) were female. BCVA was significantly lower in the patients with PDR and NPDR in comparison with NDR and normal subjects.

Mean FBS was 209.85 ± 85.6 mg/dl in the diabetic patients. Mean diabetes mellitus (DM) duration in diabetic patients was 12.7 ± 6.3 years. The study included 40 eyes in the control group and 148 eyes in the diabetic group. Based on the DR severity scale, the diabetic group had 39 (26.4%) eyes with NDR, 41 (27.7%) eyes with mild to moderate NPDR, 25 (16.9%) eyes with severe NPDR, 26 (17.6%) with early PDR and 17 (11.5%) eyes with high-risk characteristic PDR. Diabetic macular edema (DME) was present in 22 (34.4%) of NPDR and 16 (35.6%) of PDR groups. Table 1 presents the baseline characteristics of the participants.

**Table 1:** Demographic characteristics of normal subjects and subtypes of diabetic retinopathy (*N* = 188)

Groups	NL (40 eyes) (M ± SD)	NDR (39eyes) (M ± SD)	NPDR (64eyes) (M ± SD)	PDR (45eyes) (M ± SD)	***P***-value
**Age (Y)**	50 ± 0.72	59.28 ± 1.02	58.31 ± 1.14	59.03 ± 1.68	0.003
**(%) OD**	20 (50)	19 (48.7)	33 (51.6)	25 (55.6)	0.929
**Sex-male (%)**	24 (60)	15 (38.5)	25 (39.1)	20 (44.4)	0.156
**BCVA (decimal)**	0.93 ± 0.01	0.84 ± 0.02	0.65 ± 0.02	0.62 ± 0.04	< 0.001
**The last FBS (mg/dl)**	–	158.46 ± 5.72	189.78 ± 8.38	202.12 ± 7.30	< 0.001
**Duration of diabetes mellitus (Y)**	–	12.38 ± 1.02	12.96 ± 0.78	16.54 ± 1.1	< 0.001
**Hypertension number (%)**	2 (5%)	16 (41%)	27 (42%)	17 (51.5%)	< 0.001
**Hyperlipidemia number (%)**	0 (5%)	15 (38.5%)	32 (50%)	16 48.5%)	< 0.001

**BCVA Best corrected visual acuity, *DME* Diabetic macular edema, *FBS* Fasting blood sugar, *NDR* No diabetic retinopathy, *NL* Normal, *NPDR* Nonproliferative diabetic retinopathy, *PDR* Proliferative diabetic retinopathy

The difference in BCVA (decimal), sex and age were statistically significant. Spherical equivalent, axial length or intraocular pressure did not vary significantly in the groups (*p* > 0.05). Hyperlipidemia and hypertension were more prevalent in diabetic groups than the control group (*p* < 0.001). The hypertension was under control in all groups.

### Vascular density

**Fig. 1:**
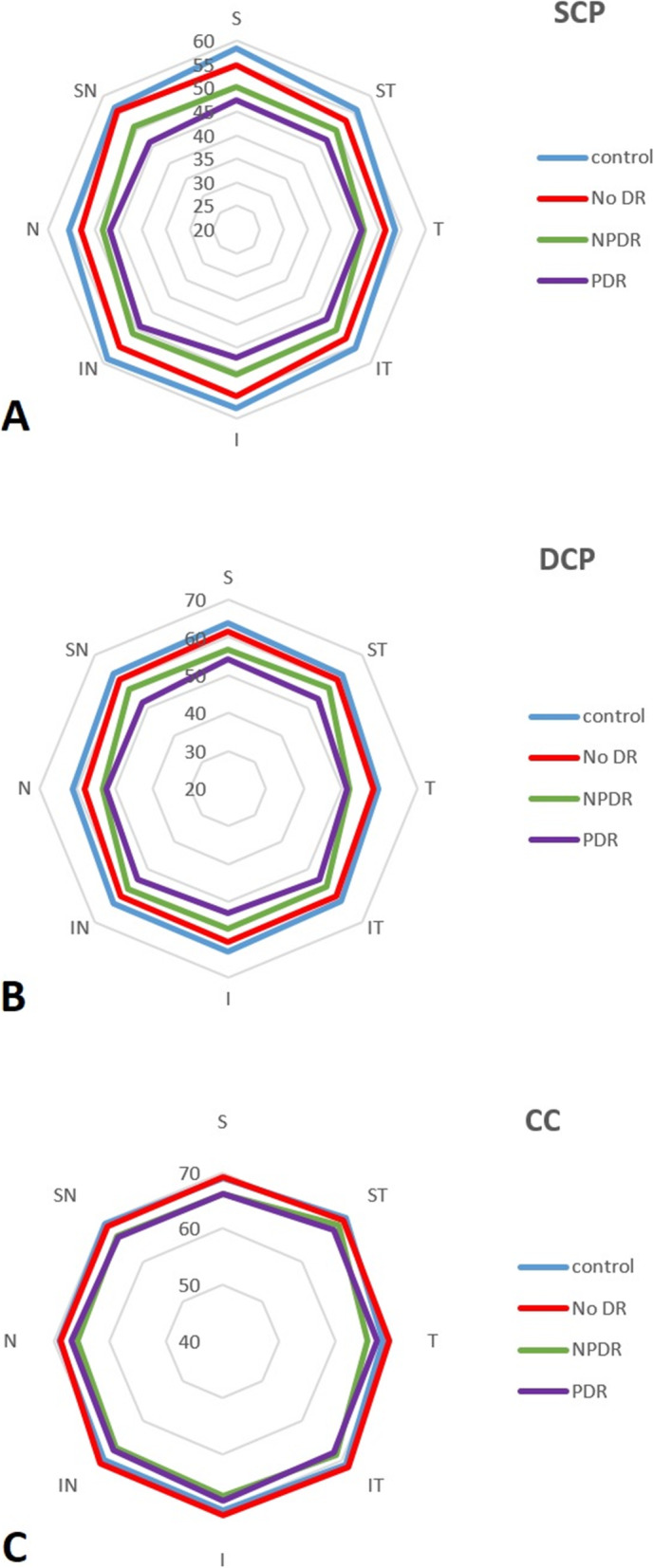
The radar diagram of choroidal thickness and choroidal volume map showing variation of the amounts taken by OCT in grid based vascular density of parafoveal area for comparison of diabetic patients in variant stages with normal eyes. *NDR: No diabetic retinopathy, NL: Normal, NPDR: Nonproliferative diabetic retinopathy, PDR: Proliferative diabetic retinopathy. ** I: Inferior, IN: inferonasal, N: Nasal, SN: Superonasal, S: superior, ST: Superotemporal, T: Temporal

Tables 2, 3, and 4 show VD in SCP, DCP, and CC, respectively. Figure 1 illustrates the distribution of grid based VD in the SCP, DCP, and CC in a radar plot. Statistically significant differences were observed in VD at various subsegments among normal control subjects and different stages of DR. All the VD amounts in SCP and DCP had abnormal distribution in the *Kolmogorov–Smirnov* test. The trend toward lower amount of VD (median) from normal subjects to PDR patients in macular area and all various subsegments was notable in both SCP and DCP. There was continuous and significant decrease from the normal cases to the NDR group in both SCP and DCP (Tables 2 and 3). The comparative tests (Mann-Whitney U test) showed that in all studied subsegments the decrease in the amounts by stepping from one stage to the others was statistically significant (*P* < 0.05). The exception was the foveal VD that was not significantly changed when comparing NDR with NPDR and NPDR with PDR in both SCP and DCP.

**Table 2:** Vascular density in superficial capillary network (SCN) of normal subjects and subtypes of diabetic retinopathy (*N* = 188)

Groups VD/ Reference group	NL (40 eyes) Median (range) (%)	NDR (39eyes) Median (range) (%)	NPDR (64eyes) Median (range) (%)	PDR (45eyes) Median (range) (%)	*P*-value Kruskal –Wallis test
**Whole image** **Normal** **NDR** **NPDR**	54.65 (47.00–50.04)	51.90 (42.40–56.28) < 0.001*	48.05 (35.07–55.78) < 0.001* < 0.001*	45.50 (33.74–50.48) < 0.001* < 0.001* < 0.001*	< 0.001
**Fovea** **Normal** **NDR** **NPDR**	29.17 (23.94–41.86)	26.26 (20.77–34.20) 0.013*	27.07 (16.11–38.10) 0.033* 0.859*	24.00 (17.68–36.14) < 0.001* 0.045* 0.062*	0.001
**Para-fovea** **Normal** **NDR** **NPDR**	57.37 (50.09–62.24)	54.54 (42.74–59.47) < 0.001*	49.96 (36.69–57.81) < 0.001* < 0.001*	47.24 (35.77–52.32) < 0.001* < 0.001* < 0.001*	< 0.001
**Superior-hemi** **Normal** **NDR** **NPDR**	57.25 (48.60–62.67)	54.03 (44.06–59.20) < 0.001*	49.92 (34.64–57.05) < 0.001* < 0.001*	46.67 (33.59–53.64) < 0.001* < 0.001* < 0.001*	< 0.001
**Inferior-hemi** **Normal** **NDR** **NPDR**	57.39 (49.50–62.60)	54.13 (41.44–59.73) < 0.001*	49.70 (38.70–58.55) < 0.001* < 0.001*	46.82 (38.06–53.41) < 0.001* < 0.001* < 0.001*	< 0.001
**Temporal** **Normal** **NDR** **NPDR**	55.76 (48.17–60.28)	53.57 (39.58–58.12) 0.001*	48.62 (36.52–57.70) < 0.001* < 0.001*	46.17 (37.79–52.46) < 0.001* < 0.001* < 0.001*	< 0.001
**Superior** **Normal** **NDR** **NPDR**	58.50 (49.62–63.98)	55.57 (45.22–59.62) < 0.001*	50.58 (36.96–57.50) < 0.001* < 0.001*	46.25 (34.26–53.65) < 0.001* < 0.001* 0.001*	< 0.001
**Nasal** **Normal** **NDR** **NPDR**	57.13 (49.50–62.64)	53.44 (44.22–58.51) < 0.001*	49.31 (33.37–57.84) < 0.001* 0.001*	46.34 (31.09–52.93) < 0.001* < 0.001* 0.002*	< 0.001
**Inferior** **Normal** **NDR** **NPDR**	57.87 (48.20–63.75)	55.06 (42.01–61.70) < 0.001*	50.35 (39.90–59.36) < 0.001* < 0.001*	47.34 (38.26–54.97) < 0.001* < 0.001* < 0.001*	0.043

*NDR* No diabetic retinopathy, *NL* Normal, *NPDR* Nonproliferative diabetic retinopathy, *PDR* Proliferative diabetic retinopathy

* Mann- Whitney test

**Table 3:** Vascular density in deep capillary network (DCN) in normal subjects and subtypes of diabetic retinopathy (*N* = 188)

Groups VD/ Reference group	NL (40 eyes) Median (range) (%)	NDR (39eyes) Median (range) (%)	NPDR (64eyes) Median (range) (%)	PDR (33eyes) Median (range) (%)	***P***-value Kruskal –Wallis test
**Whole image** **Normal** **NDR** **NPDR**	60.42 (47.00–64.55)	58.05 (51.34–60.64) < 0.001*	53.84 (41.34–60.67) < 0.001* < 0.001*	51.24 (42.50–57.58) < 0.001* < 0.001* < 0.001*	< 0.001
**Fovea** **Normal** **NDR** **NPDR**	31.24 (20.30–42.32)	27.72 (20.28–35.46) 0.001*	27.14 (15.48–40.50) < 0.001* 0.225	24.94 (12.19–39.30) < 0.001* 0.050* 0.362*	< 0.001
**Para-fovea** **Normal** **NDR** **NPDR**	62.89 (50.10–67.22)	60.77 (52.90–63.51) < 0.001*	56.49 (42.94–62.75) < 0.001* < 0.001*	54.16 (42.88–59.89) < 0.001* < 0.001* 0.001*	< 0.001
**Superior hemi** **Normal** **NDR** **NPDR**	63.45 (50.80–67.76)	61.02 (52.40–64.34) < 0.001*	56.14 (44.02–62.61) < 0.001* < 0.001*	55.33 (41.19–61.91) < 0.001* < 0.001* 0.026*	< 0.001
**Inferior hemi** **Normal** **NDR** **NPDR**	62.91 (49.50–66.68)	60.51 (52.17–63.94) < 0.001*	55.96 (40.75–63.36) < 0.001* 0.001*	54.07 (43.65–60.35) < 0.001* < 0.001* < 0.001*	< 0.001
**Temporal** **Normal** **NDR** **NPDR**	61.49 (48.50–64.82)	61.04 (49.90–63.36) 0.011*	53.33 (32.32–61.74) < 0.001* < 0.001*	53.31 (42.70–60.56) < 0.001* < 0.001* 0.004*	< 0.001
**Superior** **Normal** **NDR** **NPDR**	64.82 (51.20–69.15)	62.09 (53.30–65.34) < 0.001*	58.62 (45.35–63.41) < 0.001* < 0.001*	55.60 (42.14–60.70) < 0.001* < 0.001* 0.005*	< 0.001
**Nasal** **Normal** **NDR** **NPDR**	62.56 (52.90–67.02)	59.17 (52.40–62.53) < 0.001*	55.64 (35.39–63.44) < 0.001* 0.001*	53.48 (39.92–59.70) < 0.001* < 0.001* 0.020*	< 0.001
**Inferior** **Normal** **NDR** **NPDR**	63.76 (48.20–67.86)	62.09 (52.23–65.59) < 0.001*	56.76 (43.45–64.21) < 0.001* 0.013*	54.06 (44.73–61.92) < 0.001* < 0.001* < 0.001*	< 0.001

NDR: No diabetic retinopathy, NL: Normal, NPDR: Nonproliferative diabetic retinopathy, PDR: Proliferative diabetic retinopathy

*Mann- Whitney test

**Table 4:** Choriocapillaris (CC) vascular density in different study groups (ANOVA) normal participants and diabetic patients. Post Hoc analysis of subfields choroidal thickness of the studied participants (*N* = 188)

Groups VD/ reference group	NL (40 eyes) (M ± SD) (%)	NDR (39eyes) (M ± SD) (%)	NPDR (64eyes) (M ± SD) (%)	PDR (33eyes) (M ± SD) (%)	*P*-value ANOVA
**Whole image** **Normal** **NDR** **NPDR**	69.82 ± 4.02	70.01 ± 2.90 0.949*	67.06 ± 5.52 < 0.001* < 0.001*	67.25 ± 4.66 < 0.001* 0.003* **0.950**	< 0.001
**Fovea** **Normal** **NDR** **NPDR**	71.39 ± 3.97	71.50 ± 3.80 0.996	68.32 ± 5.16 < 0.001* 0.001*	68.98 ± 5.29 0.001* 0.003* 1.000	< 0.001
**Para-fovea** **Normal** **NDR** **NPDR**	69.36 ± 4.54	69.52 ± 2.97 O.980*	66.37 ± 5.89 < 0.001* < 0.001*	67.05 ± 4.88 0.002* 0.008* 0.798*	< 0.001
**Superior hemi** **Normal** **NDR** **NPDR**	69.92 ± 4.12	69.72 ± 2.94 0.914*	66.74 ± 5.74 < 0.001* < 0.001*	67.39 ± 4.25 0.011* 0.074* 0.437*	< 0.001
**Inferior hemi** **Normal** **NDR** **NPDR**	69.70 ± 4.06	70.28 ± 3.12 0.993*	67.16 ± 5.58 < 0.001* < 0.001*	67.70 ± 5.18 0.004* 0.002* 0.991*	< 0.001
**Temporal** **Normal** **NDR** **NPDR**	69.25 ± 5.79	69.66 ± 3.46 1.000*	66.66 ± 6.23 0.002* 0.002*	67.88 ± 6.13 0.020* 0.018* 0.964*	< 0.001
**Superior** **Normal** **NDR** **NPDR**	69.19 ± 4.52	69.19 ± 3.15 0.984*	66.66 ± 6.14 < 0.001* 0.001*	66.92 ± 4.91 0.042* 0.107* 0.488*	< 0.001
**Nasal** **Normal** **NDR** **NPDR**	69.34 ± 4.56	68.78 ± 3.63 0.785*	65.84 ± 7.10 < 0.001* 0.001*	66.92 ± 4.33 0.025* 0.236* 0.382*	< 0.001
**Inferior** **Normal** **NDR** **NPDR**	69.54 ± 4.32	70.90 ± 3.01 0.933*	66.78 ± 6.38 0.003* < 0.001*	67.33 ± 5.34 0.010* 0.001* 0.999*	< 0.001

NDR: No diabetic retinopathy, NL: Normal, NPDR: Nonproliferative diabetic retinopathy, PDR: Proliferative diabetic retinopathy

*Tuckey HSD test

**Fig. 2:**
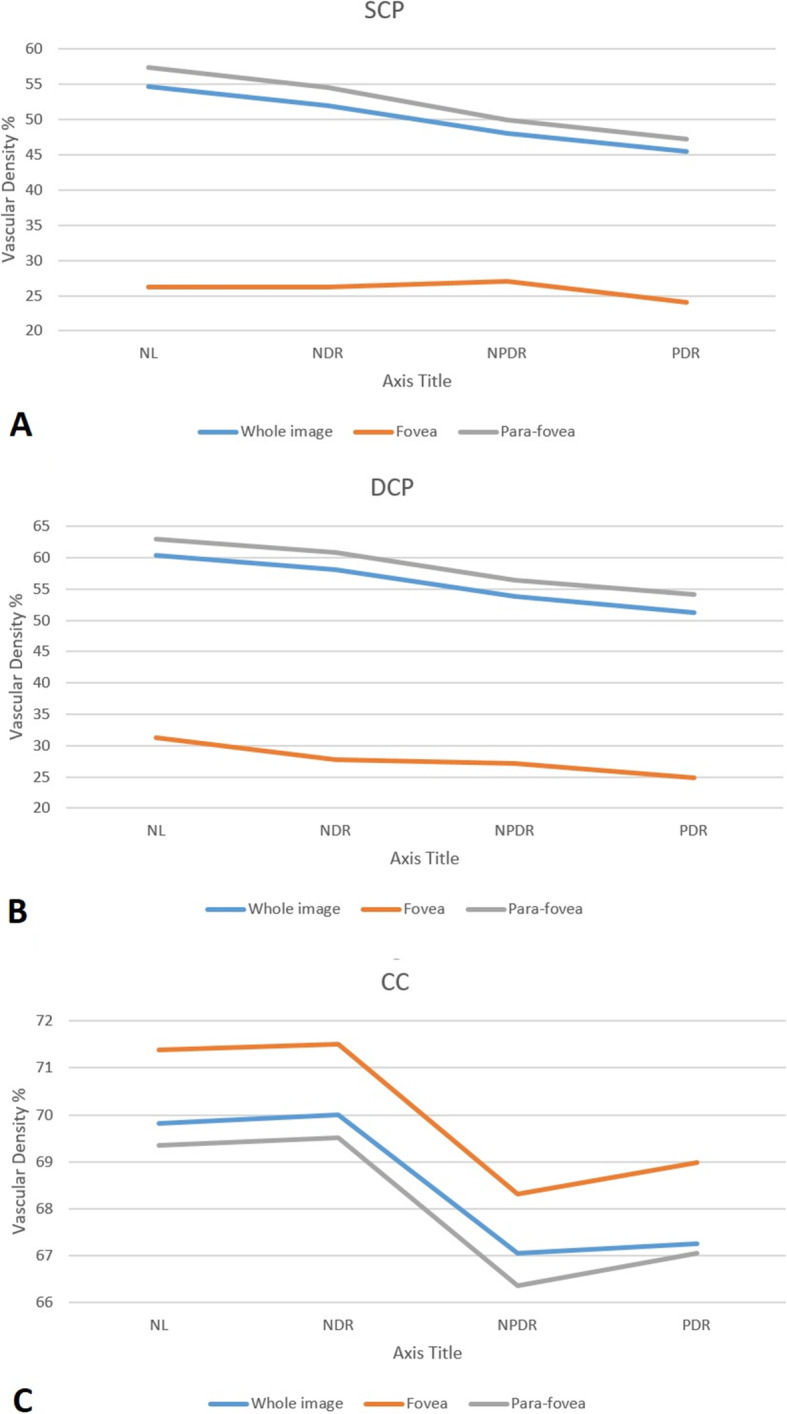
The diagram of vascular density changes from previous stage (per percentage) in 3 layers of SCP, DCP, and CC. * CC: Choriocapillaris, DCP: Deep capillary plexus, NDR: No diabetic retinopathy, NL: Normal, NPDR: Nonproliferative diabetic retinopathy, PDR: Proliferative diabetic retinopathy, SCP: Superior capillary plexus. ** I: Inferior, IN: inferonasal, N: Nasal, SN: Superonasal, S: superior, ST: Superotemporal, T: Temporal

We have seen a trend of fluctuation in the VD of SCP and DCP in the whole image and foveal region, but a steady slight decrease from NL to the PDR level is evident, which is more pronounced in the NPDR stage in the parafoveal region. (Fig. 2, Table 5).

**Table 5:** Choriocapillaris thickness changes per percentage compared to the previous stage

Percentage of changes	NDR–NL/NL (%)	NPDR-NDR /NDR (%)	PDR-NPDR/NPDR (%)
**SCP Whole image**	−5.1	−7.3	−5.4
**SCP Fovea**	−9.9	3.0	−11.4
**SCP Para-fovea**	−5.1	−8.3	−5.6
**DCP Whole image**	−3.8	1.2	−12.9
**DCP Fovea**	−11.2	−2.2	−8.1
**DCP Para-fovea**	−3.3	−7.1	−4.1
**CC Whole image**	0.3	−4.1	0.3
**CC Fovea**	0.1	−4.5	1.0
**CC Para-fovea**	0.1	−4.5	1.1

*NDR* No diabetic retinopathy, *NL* Normal, *NPDR* Nonproliferative diabetic retinopathy, *PDR* Proliferative diabetic retinopathy

In the CC the amounts of VD were normally distributed and the amount of the changes were statistically significant (ANOVA) (Table 4). Post-hoc analysis (Tuckey test) revealed no statistically significant changes between normal cases and the patients with NDR (*P* > 0.05). The VD of CC under fovea and all studied subsegments increased in NDR cases and then in NPDR and PDR cases decreased to less than those in normal cases. This comparison with NDR showed significant changes in the NPDR group in all subsegments.

### Correlations of VD

In order to determine the association of VD and other variables univariate analysis was performed for diabetic patients. Age was negatively correlated with parafoveal VD at SCP (r: -0.189, *P* = 0.015).

Systolic blood pressure was weakly inversely correlated with *WI-MVD* in CC (r: -0.155, *P* = 0.034) but not with VD in other segments. FBS was inversely correlated with WI-VD in SCP, DCP (r: -0.50, *P* < 0.001; r: -0.460, *P* < 0.001; and r: -0.222, *P* = 0.002; respectively) and foveal VD in SCP, DCP (r: -0.264, *P* < 0.001; r: -0.371, *P* < 0.001; respectively) but had weak negative correlation with foveal VD at CC layer (r: -0.141, *P* = 0.056).

DM duration in patients was also a significant factor in association with VD. It had inverse correlation with WI-VD in SCP, DCP, and CC (r: -0.354, *P* < 0.001; r: -0.352, *P* < 0.001; r: -0.106, *P* = 0.002; respectively). It was slightly correlated only with foveal VD in DCP (r: -0.263, *P* < 0.001).

Partial correlation after adjusting for the effect of age and sex showed that in diabetic patient BCVA was not correlated with the foveal VD at SCP. There was a positive correlation between BCVA and WI-VD in SCP, DCP, and CC (r: 0.497, *P* < 0.001; r: 0.505, *P* < 0.001, r: 0.233, *P* = 0.0021; respectively), foveal VD in DCP, and CC (r: 0.171, *P* = 0.023; r: 0.354, *P* < 0.001; respectively) and parafoveal VD in SCP, DCP, and CC (r: 0.542, *P* < 0.001; r: 0.520, *P* < 0.001, r: 0.354, *P* < 0.001; respectively).

In linear regression analysis, it seems that in diabetic patients after adjusting for sex, age, duration of DM, presence of DME, blood pressure and FBS in the model, among foveal VD in 3 layers, only VD of CC was significantly correlated with BCVA (B: 0.40, *P* < 0.001). This test for WI-VD in all 3 layers showed correlation with VD of SCP (B: 0.26, *P* = 0.052). In the same model for parafoveal VD, only SCP showed the correlation (B: 0.37, *P* = 0.007). Applying two significant factors, considering the collinearity, the test showed that irrespective to the staging of the DR, superficial parafoveal VD (B: 0.35, *P* < 0.001) and VD of foveal CC (B: 0.26, *P* = 0.001) had the most effect on the BCVA. Adding the staging of the DR (B: -0.25, *P* = 0.007), showed similar results.

### DME

In diabetes categories, we observed that all sub-fields had reduced VD in DME patients (all *P* < 0.05), except for foveal DCP VD (*P* = 0.716). These were far less than the amounts in the healthy control eyes (P < 0.05).

## Discussion

This study, using the commercially available OCTA method, showed lower levels of VD in the macular region and all different sub-segments of SCP and DCP in diabetic patients compared to normal controls. With the exception of SCP foveal VD and DCP WI-VD, there was a steady and substantial reduction of VD in SCP and DCP from normal cases to NDR, NDR to NPDR, and NPDR to PDR groups. In the CC, the VD decreased significantly in NPDR stage and then rose marginally (not statistically significant) in the PDR stage but never reached the normal quantities. BCVA was specifically associated with parafoveal SCP and fovea CC VD. In DME patients, VD of all subfields with the exception of foveal DCP was reduced levels when compared with those without DME.

### VD in retinal microvessels

The current study showed lower macular SCP and DCP VD levels in patients with diabetes relative to standard controls. Though methodologically different, previous studies have shown decreased parafoveal VD in DR without considering the DR staging [18, 36–39]. But our results for fovea and WI-VD in SCP and DCP are different from their results. Some qualitative or quantitative studies showed increased non-perfused areas from normal cases to the higher stages of the DR. [18, 35, 37, 38, 40]

After adjusting for hypertension, age, sex, and duration of DM our research showed that SCP and DCP VD in control eyes were higher than that in NDR group. Nesper et al. obtained the same result by adjusting to the hypertension. This finding supports VD modifications to be a potential biomarker of DR development.

The retinal vascular changes in diabetic patients including leukostasis and sluggish circulation [41–43], excitotoxic damage to glial cells [44], impaired neurovascular coupling and hypoperfusion [45] and endothelial cell and pericytes loss, may trigger capillary occlusion and hypoxia at some point [39, 40]. This indicates that slower capillary blood flow below the lowest detection level for retinal vasculature in OCTA will result in undetectable vessels in the earlier phase of diabetic retinopathy as NDR.

Unlike our result, Nesper et al. showed the strongest correlation between the DCP vessels density and with DR severity [35]. Some qualitative studies have shown a more defective VD of DCP in NDR [20], unlike our quantitative findings of a consistent decrease of VD in both SCP and DCP. Previous reports have found microaneurysms to be present in a larger extent in DCP than in the superficial plexus [41, 42, 46, 47].

The contradictory findings found in the studies may be due to different imaging and measurement techniques, methodological differences, the various phases of the disease being investigated and the varying length of DM in the studies.

### VD in CC

Some studies described diabetic choroidopathy in histology, electron microscopy, and FA and ICGA studies in the earlier stages of DR. [44, 48–51] Another study showed significant decrease in posterior ciliary arteries flow, using Color Doppler imaging, in patients with NPDR [20].

This study provides new information on CC alterations that occur in the course of DR stages from NDR to PDR. Consistent with our series, Forte et al. showed that in CC, the VD was not more different in type 1 and type 2 DM with NDR than controls in different subsegments [36].

The VD of CC was substantially reduced in NPDR compared to NDR in all subsegments and remained unchanged from NPDR to PDR (Fig. 2, Table 3). The severity of VD changes in SCP and DCP was apparently correlated with CC alteration (*P* > 0.05).

In vivo study on GK rats (a rat model of Type 2 diabetes) showed that, the VD of CC was reduced. This finding is consistent with the CC degeneration found in humans with T2D, either by post-mortem or OCT-angiography (OCTA) specimens [52–54].

Pericyte loss via vascular endothelial receptor 1 (VEGFR1), nitric oxide and reactive oxygen species identified in GK rats [55], triggered by hypoxia and/or inflammation [56], appears to be causing capillary degeneration and subsequent thinning during NPDR phase. This may contribute to a subsequent ischemic cascade involving CC, RPE and the outer retina propagating diabetic retinopathy.

### Correlation of VD and visual acuity

After adjusting to several conflicting factors, this study found that the vision was directly associated with parafoveal VD at SCP and foveal VD at CC. The result was independent of the stage of the DR. Dissimilar to our result, previous researches showed that VD of DCP has more impact on the BCVA than SCP [57].

In diabetic patients, the reasons for reduced VA include macular ischemia [58], photoreceptor dysfunction [59] and accumulated subfoveal hard exudates [60]. There is a correlation between retinal nonperfusion and the integrity of the photoreceptors [35, 61]. There was a strong correlation between external limiting membrane (ELM) and ellipsoid zone (EZ) integrity in DME patients [62].

Using oxygen-sensitive microelectrodes and oximetry, Linsenmeier and Zhang discovered that the choroidal and retinal circulation could supply the metabolic demand of photoreceptors in various proportions under both dark and light conditions [63]. It is important to know that, while most of the outer retina including photoreceptor is nourished with choroidal circulation, the contribution of the retinal circulation to photoreceptor metabolism is modest [63]. They calculated that the photoreceptor demand, met by the retinal circulation, was different in dark and lighted conditions (10 to 18% in darkness) [63].

Nesper et al. observed changes in capillary dropout areas in the DCP, indicating that DCP integrity is required for the metabolism of the photoreceptor [64].

Recently, by using spectral-domain OCT (SD-OCT), the concept of disorganization of retinal inner layers (DRIL) has been described to characterize retinal thinning with the loss of identifiable borders between retinal cellular layers in the background of capillary non-perfusion [65, 66]. Areas of DRIL were significantly correlated with disturbance of the photoreceptor (*P* = 0.035) relative to adjacent DRIL-free areas [66].

The decrease in VD in the SCP was significantly associated with the decrease in inner retinal thickness in DR, a notorious finding in mild DR, which may contribute to DRIL in the case of more serious capillary dropout [65]. Conversely, there was no association between the decrease in VD in the DCP and the thickness of the inner nuclear / outer plexiform layer (INL / OPL). The explanation for this disparity, also seen in healthy eyes, is unknown [66–70]. May be the SCP is much important for the inner layer nourishment. Moein et al. reported capillary flow impairment in the SCP on OCTA may be a predictor of visual acuity in patients with DRIL [71]. It is believed that the SCP, whose vascular supply is mainly arterial [72], has a standardized measurement, and is better to reveal ischemia data than the DCP [73].

### DME

Macular edema can develop at any stage of DR. We found that in all subfields, the diabetic patients with DME had less VD than the patients without DME, except for the foveal VD at DCP. Tang et al. did not detect any statistically significant correlation between OCTA metrics and the existence of DME on SCP, incompatible with our findings [74]. Lee et al. have recently shown that DME eyes only have lower VD at DCP relative to DME-free eyes [75].

A limitation of this study was a modest age difference between control group and other groups, although in all stages of the analytic part of the study, the age matching was performed. Some baseline data as smoking and type of prescription have not been considered in the investigation, which may influence our conclusions. The small field of OCTA imaging and the use of both eyes in the analysis were other limitations. The automated programmed algorithm in the AngioVue system only segments two retinal capillary plexuses: the SCP and the DCP, and the middle capillary plexus (MCP) that is defined by swept source OCT could not be calculated.

Despite previous similar study [35], the advantage of present study was the inclusion of treatment naïve cases and an almost large sample size as well as the use of statistical models with adjustments for covariates.

## Conclusions

In assumption, our study showed that VD in OCTA is correlated significantly and linearly with disease severity in eyes with DR. The results support VD of retinal vessels and CC to be a potential surrogate for DR before clinical signs development. We introduced parafoveal VD of SCP and foveal VD of CC as biomarkers to predict the VA loss in the diabetic patient. As a noninvasive and rapidly acquired technique, OCTA may be a tool for early detection of microvascular abnormalities in the retina and choroid, elucidating the pathogenesis of retinopathy, and treatment response monitoring in patients with diabetes.

## Data Availability

The datasets used and/or analyzed during the current study are available from the corresponding author on reasonable request.

## Abbreviations

CC: choriocapillaris
CMT: central macular thickness
DR: diabetic retinopathy
DME: diabetic macular edema
MCP: middle capillary plexus
NPDR: nonproliferative diabetic retinopathy
OCTA: optical coherence tomography angiography
PDR: proliferative diabetic retinopathy
SD OCTA: spectral domain optical coherence tomography angiography
DCP: deep capillary plexus
SCP: superficial capillary plexus
VD: vascular density
VEGF: vascular endothelial growth factor
WI: whole image
